# Gender differences in first and secondhand smoke exposure, spirometric lung function and cardiometabolic health in the old order Amish: A novel population without female smoking

**DOI:** 10.1371/journal.pone.0174354

**Published:** 2017-03-31

**Authors:** Robert M. Reed, Mark T. Dransfield, Michael Eberlein, Michael Miller, Giora Netzer, Mary Pavlovich, Toni I. Pollin, Steven M. Scharf, Alan R. Shuldiner, Don Sin, Braxton D. Mitchell

**Affiliations:** 1 University of Maryland School of Medicine, Division of Pulmonary and Critical Care Medicine, Baltimore, Maryland, United States of America; 2 University of Alabama School of Medicine, Division of Pulmonary and Critical Care Medicine, Birmingham, Alabama, United States of America; 3 University of Iowa School of Medicine, Division of Pulmonary and Critical Care Medicine, Iowa City, Iowa, United States of America; 4 University of Maryland School of Medicine, Division of Endocrinology, Diabetes, and Nutrition, Baltimore, Maryland, United States of America; 5 Department of Veterans Affairs and Veterans Affairs Medical Center Baltimore Geriatric Research Education and Clinical Center (GRECC), Baltimore, Maryland, United States of America; 6 University of Maryland School of Medicine, Department of Epidemiology and Public Health, Baltimore, Maryland, United States of America; 7 University of British Columbia Respiratory Medicine; Vancouver, British Columbia, Canada; Dartmouth College Geisel School of Medicine, UNITED STATES

## Abstract

Due to their relatively homogeneous lifestyle and living environment, the Amish offer a novel opportunity to study the health associations of tobacco smoke exposure, particularly secondhand smoke. We hypothesized that secondhand smoke exposure is associated with worse pulmonary and cardiometabolic health. We examined cross-sectional data on 3568 Amish study participants, including tobacco use and secondhand smoke exposure from family members included in the study. Thirty-four percent of Amish men reported ever smoking. Of this proportion, 64% used cigars, 46% cigarettes, and 21% pipes. Less than 1% of women reported ever smoking. Smoking was associated with lower spirometric lung function, higher body mass index, lower HDL cholesterol, higher heart rate, lower ankle-brachial index, and larger aortic diameter in men. A greater number of sources of secondhand smoke exposure (defined from the total of spouses, parents, and siblings who smoke) was associated with higher body mass index (p = 0.03) and with higher fasting glucose in men (p = 0.01), but not in women (p = 0.007 for sex*secondhand smoke interaction). Secondhand smoke exposure was also associated with reduced HDL cholesterol only in women (p = 0.002) and a lower heart rate only in men (p = 0.006). Smoking habits among the Old Order Amish are notable for the absence of female participation and a high proportion of cigar and pipe use. Smoking is associated with decreased spirometric indices of lung function and increased cardiovascular risk in this population and secondhand smoke exposure is associated with a greater burden of risk factors for cardiovascular disease. Sex differences in correlations could reflect differences in exposure patterns, mechanisms, or susceptibilities.

## Introduction

Tobacco smoking is the leading preventable cause of death in the United States, responsible for an estimated 480,320 deaths annually, including 41,280 deaths attributable to lung cancer and coronary heart disease related to secondhand smoke exposure[1]. Secondhand smoke consists of main-stream smoke (the smoke that is inhaled and then exhaled by the smoker) and side-stream smoke (smoke that wafts directly from the burning tobacco). Secondhand smoke exposure has been associated with increased risks of COPD[2], lung cancer[3], stroke[4], as well as various fetal and pediatric health issues including low birth weight and infant death[1, 5]. While uncertainty remains, emerging data suggest secondhand smoke may also contribute to diabetes[6], obesity[7], and hypertension[1, 8].

Epidemiologic issues complicate correlative studies of the effects of tobacco smoke exposure and make the Amish a population well-suited for such investigation. Socioeconomic status and educational levels are associated with both smoking behavior[9] and cardiovascular risk[10], but within the Amish these and other lifestyle factors are relatively homogeneous[11]. Another key benefit to studying secondhand smoke in the Amish is that smoking is virtually nonexistent among Amish women[12, 13]. In populations including female smokers, it is difficult to disentangle the effects of secondhand smoke from the exposures uniquely associated with maternal smoking such as intrauterine fetal exposure from a mother who smokes, high-intensity side-stream smoke exposure during infancy, and exposure through breastmilk[9], which are exposures absent in the Amish.

Cigars and pipes represent the predominant forms of tobacco smoked by the Amish[12] and these create a greater degree of side-stream smoke than do cigarettes[14], thus presenting potential for a significant burden of secondhand tobacco exposure on the family members of those who smoke. As such, the Amish represent a useful population in which to examine the associations of secondhand smoke exposure.

For these reasons, we examined the health characteristics associated with smoking patterns in a cross sectional sample of the Old Order Amish of Lancaster County to consider the hypothesis that smoke exposure in this novel population would be associated with pulmonary, cardiac, and metabolic derangements.

## Materials and methods

### Data source

This report is based on data obtained in three community surveys of cardiovascular health in the Amish between 2001 and 2015[15, 16]. All studies were conducted with participant informed consent and with approval from the Institutional Review Board of the University of Maryland.

### Inclusion criteria and definitions

Enrollment in each survey was limited to adults (age ≥18 years). The majority (n = 2683) participated in a wellness screening program offered to the community beginning in 2010 and open to all Amish adults. An additional 506 were recruited into a study that limited enrollment to age ≥30 years and excluded pregnant women[15]. The remaining 379 participated in a study of longevity conducted between 2001–2006 that enrolled participants ≥ 90 years of age, as well as their offspring and the spouses of their offspring[16]. Amish who participated in multiple studies were included only once in these analyses, preferentially using data from the wellness screening program. Diabetes was defined by self-report, use of a diabetic medication, or fasting glucose ≥7.0mmol/, and obesity was defined by a body mass index ≥30.

Smoking history was ascertained through self-report using questionnaires administered by research coordinators. “Ever smoking” was defined by either reporting any cigarette, pipe, or cigar use, or by answering affirmatively to having ever smoked regularly. All secondhand smoking analyses were limited to participants who had never smoked. Family and spousal smoking was characterized based on the self-reported smoking status of enrolled family members. We examined secondhand smoke exposure in both dichotomous (exposed/not exposed) terms as well as in ordinal terms according to a score relating to the number of family members reporting a history of smoking. For dichotomous analyses a study participant was considered exposed to secondhand smoke if either the participant’s father, husband, or brother reported himself to be a current or an ex-smoker. To quantify the number of sources of secondhand smoke, we counted the number of smokers in the nuclear family, assigning 1 point if the participant’s father smoked, 1 point if the participant’s husband smoked, and 1 point if one or more of the participant’s brothers smoked. We then generated a secondhand smoking exposure category score ranging from 0 to 3, based on the sum of these points. For this analysis, the secondhand smoke exposure category was set to missing if the smoking status of either the father or the husband of the participant was unknown.The number of parental sources of secondhand smoke exposure has previously been shown to correlate with cardiovascular measures[17].

### Assessments

Blood samples were taken while fasting and processed in CLIA (clinical laboratory improvement amendments) certified centers. Spirometry was performed in accordance with ATS standards[18] without bronchodilator, and interpreted in terms of reference values for a Caucasian cohort [19]. Vascular health was assessed using ankle brachial index (ABI) and aortic ultrasound measures on fasting individuals over age 40. Mean ABI was obtained by averaging measures from the left and right side. Aortic ultrasound measures were performed using a C4-7 probe with a Vivid e, GE laptop ultrasound device. Longitudinal and transverse images were recorded at a minimum of 7 locations along the descending aorta, and the maximum outer-to-outer measure was identified.

### Statistical analysis

Data are presented as counts and percentages or as medians with interquartile range (IQR). In order to control for non-independence of observations due to family clustering effects, we performed regression analyses using the linear mixed model software as implemented in MMAP (mixed models analysis for pedigrees), which accounts for relatedness of individuals (family structure) through inclusion of relationship matrix as a random effect[19]. We also used MMAP to examine interaction factors for sex in linear models to complement sex-stratified analyses. All other statistical analyses were performed using STATA 14 SC software (StataCorp-LP, College Station, TX). Despite examination of multiple outcome variables a Bonferroni correction was considered unnecessary based on suggested criteria for use [20]. For all analyses, two-tailed p<0.05 was considered significant.

## Results

The study group included 3568 adult Amish individuals, of whom 43% were men. Four percent of the group were diabetic and 25% were obese. A history of having ever smoked was reported by 519 (34%) men, and 2 (<1%) women. Of men who reported having ever smoked, 46% also reported current smoking. Among smokers, cigar smoking was most common (64%), followed by cigarette (46%), and then pipe (21%). Median cigarette pack-years smoked (one pack a day for a year) was 4.5 (IQR 1.5 to 11.6), cigar-years (1 cigar a day for a year) 65.8 (IQR 15.7 to 180), and pipe-years (one pipe bowl a day for a year) 47.6 (IQR 22.3 to 86.0). Heavy cigarette smoking was uncommon, with 19% of smokers reporting >10 pack-years of lifetime cigarette smoking. Using previously proposed cigarette-per day equivalency estimates based on nicotine content for pipes and the small cigars most commonly smoked among the Lancaster Amish[12], a >10 pack-year “equivalent” was reported by 22% of cigar smokers and 58% of pipe smokers.

### Firsthand tobacco exposure in men

A history of having ever smoked was associated with older age (median 54 vs. 44 years, p<0.0001), and higher BMI (27 vs.25, p<0.0001) (**Table 1**). Smoking was associated with lower FEV1/FVC%predicted (104 vs. 106%, p<0.0002), and this difference was independent of BMI. The association between higher BMI and smoking persisted after controlling for age differences, and appeared to be a result of both higher weights (p<0.0001) as well as shorter statures (p = 0.005). After controlling for age and BMI, smoking was associated with lower high density lipoprotein cholesterol (HDL-C) levels (p<0.001), higher triglyceride levels, higher resting heart, lower mean ankle-brachial index, and higher aortic size.

**Table 1:** Health Related Correlates of Smoking in Old Order Amish from Lancaster County

	Never Smoking Men (n = 1021)	Ever Smoking Men (n = 519)	P	Never Smoking Women (n = 2026)
Age at evaluation (yrs)	44 (33 to 58)	54 (40 to 65)	<0.0001	44 (31 to 59)
BMI (kg/m^2^)	25 (23 to 28)	27 (24 to 30)	<0.0001*	27 (23 to 31)
Height (cm)	172 (169 to 177)	173 (168 to 177)	0.005*	161 (157 to 165)
Weight (kg)	75.6 (68.4 to 84.5)	80.4 (72.6 to 89.6)	<0.0001*	68.8 (60.0 to 79.0)
Waist to Hip Ratio	0.92 (0.88 to 0.96)	0.94 (0.90 to 0.98)	0.0001*	0.83 (0.79 to 0.87)
FEV1 (% predicted)	95 (82 to 105)	92 (78 to 105)	0.1†	91 (79 to 101)
FVC (% predicted)	89 (79 to 98)	90 (80 to 101)	0.4†	88 (78 to 99)
FEV1/FVC (%predicted)	106 (99 to 113)	104 (95 to 110)	0.0002†	104 (96 to 110)
LDL-C (mmol/L)	3.39 (2.82 to 4.06)	3.47 (2.84 to 4.06)	0.2‡	3.28 (2.59 to 4.06)
HDL-C (mmol/L)	1.45 (1.22 to 1.68)	1.29 (1.09 to 1.53)	0.0003‡	1.63 (1.40 to 1.94)
TG (mmol/L)	0.64 (0.49 to 0.88)	0.81 (0.59 to 1.16)	0.00003‡	0.73 (0.54 to 1.10)
Heart Rate by EKG	62 (56 to 68)	64 (58 to 72)	<0.0001‡	67 (62 to 73)
Fasting glucose (mmol/L)	4.8 (4.5 to 5.1)	4.9 (4.6 to 5.3)	0.5‡	4.7 (4.4 to 5.1)
SBP (mmHg)	110 (103 to 120)	114 (106 to 125)	0.95‡	109 (101 to 122)
Mean ABI (n = 797)	1.25 (1.17 to 1.33)	1.20 (1.12 to 1.30)	0.0005‡	1.17 (1.10 to 1.26)
Aorta (cm) (n = 595)	2.1 (1.9 to 2.4)	2.2 (1.9 to 2.5)	0.046‡	1.9 (1.7 to 2.2)

Data are expressed as medians (IQR) or as percentages. Unadjusted P values were obtained using Wilcoxon rank sum. Adjusted p values were obtained using linear regression and were limited to men.

*Adjusted for family structure and age.

**†**Adjusted for family structure.

‡Adjusted for family structure, age, and BMI.

ABI: ankle-brachial index; BMI: body mass index; DBP: diastolic blood pressure; EKG: electrocardiogram; FEV1: forced expiratory volume in 1 second; FVC: forced vital capacity; LDL-C: low density lipoprotein cholesterol; HDL-C: high density lipoprotein cholesterol; SBP, systolic blood pressure; TG; triglycerides.

### Secondhand tobacco exposure: Dichotomous approach

Evaluation of secondhand smoke exposure was evaluated in dichotomous terms separately in never-smoking men and women (data not shown). Differences in spirometric lung function associated with secondhand smoke were not appreciable in men, but were detected in women. The magnitude of these differences was small, with a mean difference (assessed by Student’s T-test) in FEV1% of 2.7% (p = 0.008) and FVC% of 2.8% (p = 0.002). These differences persisted after adjustment for BMI. Analyses adjusted for age and BMI also showed secondhand smoke to be associated with significantly lower HDL-C in women (p = 0.04), but not men (p = 0.98).

### Secondhand tobacco exposure: Ordinal approach

Characteristics of nonsmokers according to number of secondhand smoking sources are presented in **Table 2**. Forty percent of women and 48% of never-smoking men had no family member identified as a source of smoke exposure. Potential family sources of exposure in women was primarily from fathers (47%), followed by siblings (23%) and spouses (20%). Of never-smoking men, 39% had fathers with a smoking history and 26% had siblings with a smoking history. No men had more than two sources because none of their spouses smoked. The patterns of exposure in this subgroup were representative of the exposures in the entire group. In women, secondhand smoke exposure was associated with older age (p < 0.0001) and lower HDL cholesterol (age-adjusted p = 0.002). In men, secondhand smoke exposure was associated with older age (p < 0.0001), higher heart rate (age-adjusted p = 0.006), and higher fasting glucose (age-adjusted p = 0.01).

**Table 2:** Health Related Correlates To Categories of Passive Smoking in Old Order Amish Study Participants.

	No smoke exposure	1 source of secondhand smoke exposure	2 sources of secondhand smoke exposure	3 sources of secondhand smoke exposure	P*
**Women**					
	n = 246	n = 223	n = 124	n = 29	
Age at evaluation (yrs)	28 (21 to 38)	36 (27 to 45)	40 (34 to 49)	44 (37 to 53)	<0.0001
BMI (kg/m^2^)	23.9 (21.8 to 27.0)	25.4 (22.5 to 29.8)	26.2 (23.3 to 29.6)	26.8 (24.9 to 30.3)	0.1
Height (cm)	163 (159 to 167)	162 (159 to 165)	161 (158 to 165)	160 (158 to 165)	0.4
Weight (kg)	64.0 (57.8 to 72.0)	66.2 (59.0 to 77.2)	67.7 (60.9 to 77.5)	69.6 (63.4 to 79.0)	0.2
Waist to Hip Ratio	0.82 (0.78 to 0.86)	0.83 (0.80 to 0.87)	0.83 (0.79 to 0.88)	0.82 (0.79 to 0.87)	0.7
FEV1 (% predicted)	92 (82 to 102)	90 (78 to 100)	93 (82 to 101)	91 (78 to 100)	0.09
FVC (% predicted)	90 (80 to 101)	89 (80 to 99)	91 (81 to 98)	90 (79 to 98)	0.07
FEV1/FVC (% predicted)	103 (96 to 108)	102 (94 to 109)	101 (95 to 106)	102 (94 to 109)	0.7
LDL-C (mmol/L)	2.84 (2.33 to 3.57)	2.79 (2.35 to 3.65)	3.05 (2.53 to 3.72)	3.15 (2.79 to 3.93)	0.8
HDL-C (mmol/L)	1.71 (1.50 to 2.02)	1.63 (1.40 to 1.99)	1.60 (1.34 to 1.91)	1.63 (1.32 to 1.84)	0.002
TG (mmol/L)	0.58 (0.45 to 0.75)	0.63 (0.50 to 0.88)	0.67 (0.52 to 0.99)	0.75 (0.56 to 1.05)	0.7
Heart Rate by EKG	69 (63 to 74)	66 (60 to 73)	67 (62 to 74)	68 (63 to 75)	0.9
Fasting glucose (mmol/L)	4.50 (4.22 to 4.78)	4.56 (4.28 to 4.89)	4.67 (4.39 to 5.00)	4.83 (4.44 to 5.17)	0.99
SBP (mmHg)	103 (98 to 110)	104 (98 to 111)	107 (100 to 115)	107 (99 to 117)	0.7
DBP (mmHg)	67 (62 to 72)	67 (62 to 73)	69 (64 to 75)	71 (67 to 73)	0.9
**Men**					
	n = 264	n = 161	n = 38	n = 0	
Age at evaluation (yrs)	32 (26 to 40)	40 (31 to 51)	38 (33 to 43)		<0.0001
BMI (kg/m^2^)	24 (22 to 26)	25 (23 to 27)	25 (24 to 27)		0.2
Height (cm)	174 (171 to178)	173 (168 to 177)	173 (171 to 177)		0.8
Weight (kg)	72.6 (67.8 to 79.5)	75.4 (68.8 to 82.2)	78.0 (70.2 to 81.2)		0.1
Waist to Hip Ratio	0.90 (0.86 to 0.93)	0.91 (0.87 to 0.95)	0.93 (0.89 to 0.98)		0.2
FEV1 (% predicted)	95 (83 to 105)	97 (85 to 106)	94 (90 to 101)		0.5
FVC (% predicted)	91 (83 to 100)	88 (81 to 98)	88 (80 to 99)		0.5
FEV1/FVC (% predicted)	102 (95 to 108)	104 (97 to 108)	103 (94 to 107)		0.4
LDL-C (mmol/L)	3.13 (2.51 to 3.90)	3.31 (2.74 to 4.03)	3.57 (2.66 to 4.45)		0.8
HDL-C (mmol/L)	1.50 (1.27 to 1.73)	1.42 (1.24 to 1.71)	1.50 (1.22 to 1.68)		0.8
TG (mmol/L)	0.53 (0.41 to 0.70)	0.65 (0.49 to 0.94)	0.56 (0.46 to 0.75)		0.1
Heart Rate by EKG	63 (56 to 69)	60 (57 to 66)	56 (52 to 65)		0.006
Fasting glucose (mmol/L)	4.72 (4.44 to 4.94)	4.78 (4.56 to 5.00)	4.83 (4.67 to 5.06)		0.01
SBP (mmHg)	108 (101 to 115)	109 (102 to 116)	107 (101 to 113)		0.5
DBP (mmHg)	70 (64 to 75)	71 (65 to 76)	71 (64 to 74)		0.5

Data are expressed as medians (IQR) or as percentages.

*P values were obtained using MMAP software controlling for pedigree and age (with the exception of age and spirometric measures, which were controlled for pedigree alone).

ABI: ankle-brachial index; BMI: body mass index; DBP: diastolic blood pressure; EKG: electrocardiogram; FEV1: forced expiratory volume in 1 second; FVC: forced vital capacity; LDL-C: low density lipoprotein cholesterol; HDL-C: high density lipoprotein cholesterol; SBP: systolic blood pressure; TG: triglycerides.

**Table 3** shows the association between secondhand smoking score and cardiovascular risk factors in the combined set of men and women. With adjustment for age and sex, secondhand smoking score was significantly associated with higher BMI (p = 0.03). After adjusting for age, sex, and BMI, secondhand smoking score remained significantly associated with lower HDL-C (p = 0.045). Secondhand smoking score was also significantly associated with higher fasting glucose levels in men (age and BMI-adjusted p = 0.01), but not women (sex*secondhand smoking score interaction p = 0.007). No association was detected between secondhand smoke exposure and ABI or aortic size in either sex-stratified (data not presented) or combined set. These vascular measures were made only in participants ≥40 years of age, and analyses were limited by the relatively small sample sizes.

**Table 3:** Correlation Between Health Measures and Ordinal Categories of Passive Smoking in Old Order Amish Study Participants from Lancaster County.

	Adjusted for	β (SE)	P	Interaction P (sex*smoke exposure)
FEV1 (liters)	Age, Height, Sex, BMI	-0.03 (0.03)	0.4	0.8
FVC (liters)	Age, Height, Sex, BMI	-0.2 (0.06)	0.06	0.09
FEV1/FVC	Age, Height, Sex, BMI	0.04 (0.02)	0.052	0.02
BMI (kg/m^2^)	Sex, Age	0.4 (0.2)	0.03	0.5
Waist to Hip Ratio	Sex, Age	0.0005 (0.003)	0.8	0.1
Height (cm)	Sex, Age	-0.05 (0.3)	0.8	0.9
Weight (kg)	Sex, Age	1.07 (0.5)	0.05	0.8
HDL-C (mmol/L)	Sex, Age, BMI	-0.04 (0.02)	0.045	0.4
HDL-C (mmol/L)	Sex, Age, BMI, TG	-0.04 (0.02)	0.02	0.2
LDL-C (mmol/L)	Sex, Age, BMI	-0.02 (0.05)	0.6	0.3
TG (mmol/L)	Sex, Age, BMI	-0.002 (0.02)	0.9	0.3
Glucose (mmol/L)	Sex, Age, BMI	0.42 (0.14)	0.003	0.007
SBP (mmHg)	Sex, Age, BMI	-0.3 (0.5)	0.5	0.3
DBP (mmHg)	Sex, Age, BMI	-0.3 (0.4)	0.5	0.7
Heart Rate	Sex, Age, BMI	-3.3 (1.5)	0.02	0.07
Mean ABI (n = 342)	Sex, Age, BMI	0.001 (0.01)	0.9	0.2
Aorta (n = 273)	Sex, Age, BMI	0.02 (0.02)	0.5	0.4

N = 1056.

P values were obtained using regression methods including controls for family structure. ABI: ankle-brachial index; BMI: body mass index; DBP: diastolic blood pressure; FEV1: forced expiratory volume in 1 second; FVC: forced vital capacity; LDL-C: low density lipoprotein cholesterol; HDL-C: high density lipoprotein cholesterol; SBP: systolic blood pressure; TG: triglycerides.

## Discussion

We found that both smoking and secondhand tobacco exposures in a novel Amish population were associated with cardiovascular risk factors. Our data provide evidence supporting an association between secondhand smoke exposure and higher glucose levels, higher BMI, lower HDL-C, lower heart rates, and lower spirometric measures of lung function. These associations demonstrated notable gender differences.

### First hand smoke exposure

The Amish study group presented here included only male smokers with a relatively high frequency of cigar (64%) and pipe (21%) use. For context, smoking preferences in the general US population favor cigarettes (84% of smokers) with far fewer smokers using cigars (19% of smokers) or pipes (3% of smokers)[21]. As cigar and pipe users typically inhale less deeply, such smoking involves more side-stream rather than main-stream smoke inhalation, and thus it more closely resembles secondhand smoke than the first-hand smoking of cigarettes[14, 22]. Despite this and modest smoking habits overall, adjusted analyses demonstrated associations between smoking and detectable differences in spirometric lung function, BMI, HDL-C, and heart rate. Furthermore, smoking was associated with subclinical cardiovascular disease as evidenced by lower ankle-brachial index and larger aortic size. This supports prior assertions that a large proportion of cardiovascular risk attributable to tobacco exposure occurs at low levels of exposure[23, 24]. Prior observations have suggested that this low threshold for effect may be attributable primarily to prothrombotic effect[23, 24]. Our data would suggest a low threshold for other mechanisms as well.

### Pulmonary associations of secondhand smoke

Our study found a 2.7% lower spirometric lung function (FEV1%) associated with secondhand smoke in women. The power to detect this difference in the female group was 98%, whereas power was only 65% to detect this difference in the never-smoking men. As such, the lack of signal in men may represent a limitation of power rather than a true gender difference. Power in our study as well as in prior studies could be impaired due to issues of nondifferential misclassification bias related to ascertainment of smoke exposure, potentially resulting in both difficulty detecting effect as well as underestimation of effect size. This might particularly affect studies assessing only one source of tobacco exposure, such as spousal smoking[25–27]. The Surgeon General’s review states, “The evidence is suggestive but not sufficient to infer a causal relationship between chronic secondhand smoke exposure and a small decrement in spirometric lung function in the general population[23]” Notably, a meta-analysis involving 9 cross sectional studies reported an estimate similar to ours (-2.7%, 95%CI -4.1 to -1.2%)[28]. Our results bolster that observation and should reduce uncertainty around the association between secondhand smoke exposure and small decrements in spirometric lung function.

### Cardiometabolic associations of secondhand smoke

The cardiovascular risk associated with secondhand smoke exposure is striking and approaches that of smoking[29, 30]. Studies demonstrate a consistent 25–30% increased risk for coronary disease associated with secondhand smoke[23]. The best established mechanisms underlying this effect include endothelial dysfunction and a prothrombotic effect[23]. Our findings suggest the possibility of a significant role for mechanisms less clearly established in secondhand smoking, but recognized in association with active smoking. Such effect mediators include HDL-C and BMI.

We found lower HDL-C levels associated with secondhand smoke exposure in women only. Svendsen described a cohort of 1245 never-smoking men in whom HDL-C levels were not associated with spousal smoking status[27]. Another study described lower HDL-C in female adolescents exposed to secondhand smoke from their parents, but no such association existed in males[31]. Notably, smoking is clearly associated with reduced HDL-C levels in men[32], including in our cohort. As such, it would seem that both men and women manifest reduction in HDL-C levels in response to smoke exposure, but women may have a greater susceptibility for this particular effect. While our study design does not facilitate determination of mechanism, it is likely that the differences observed between men and women result from both differences in exposure as well as susceptibility. Differences in susceptibility are supported by biologic plausibility. Smoke exposure has been shown to influence sex hormone profiles in women[33], and sex hormone profiles in turn correlate with levels of HDL-cholesterol, independently of smoking[34].

We found more familial sources of secondhand smoke to correlate with higher BMI in both men and women without apparent differential effect according to gender. A higher BMI has been associated with secondhand smoke in a number of large studies[17, 27, 35–38]. It has been suggested that tobacco smoke is a source of a variety of “obesogens”, including polycyclic aromatic hydrocarbons, which are produced by incomplete combustion of organic materials[38]. These polycyclic aromatic hydrocarbons are produced from other sources as well, and may function synergistically with secondhand smoke to promote obesity[38].

Heart rate in our cohort was higher in smoking men, lower in men exposed to secondhand smoke, and unassociated with secondhand smoke exposure in women. Our findings in smokers reflect prior observations consistent with increased adrenergic tone associated with smoking[32]. The observation of paradoxically lower heart rates in men in and absence of association women exposed to secondhand smoke is not easily explained, although a murine model has shown estrogen to modulate chronotropic effects of nicotine[39]. Notably, the possibility of confounding attributable to use of medications that would affect the heart rate is possible but unlikely in our cohort as medication use is not common in the Amish, with rates of beta blocker use under 5%[40]. Smoking has been associated with insulin resistance and increased risk for the development of type 2 diabetes in both men and women[6, 32, 41, 42]with an apparent dose-reponse relationship. Secondhand smoke has also been associated with increased risk for type 2 diabetes[6, 42]. A large meta-analysis showed a stronger correlation between smoke exposure and elevated glucose levels in men[42], possibly suggestive of a differential susceptibility according to sex. Supporting this possibility, we found more family sources of secondhand smoke exposure to be associated with higher glucose levels in men, but not women.

### Strengths and limitations

Our methods for assessment of secondhand smoke exposure involved questioning family members about their smoking history and then extrapolating exposure through pedigree rather than questioning study participants about secondhand smoke exposure directly. While this approach mitigates recall bias, it led to some data points missing due to nonrandom inability to capture all family members (older participants were less likely to have parents enrolled) that was not possible to meaningfully address through imputation. The methods also did not permit differentiation between acute exposure versus chronic or past exposure. It is likely that the different categories of potential sources comprising the exposure score reflect exposures occuring primarily at different periods of life such that exposure from a father would be more likely to occur during childhood whereas exposure from a spouse would more likely represent a more recent or current source. Power was insufficient to meaningfully distinguish differences between these potential sources of exposure. Workplace exposure was also not captured in our data and represents a possible source of misclassification bias that would likely be nondifferential and thus bias towards the null in analyses stratified by sex, but could represent a source of differential exposure between men and women.Finally, the cross-sectional nature of our study is suceptible to survivorship bias. We would, however, expect mortality related to smoke exposure to preferentially affect those with high exposure or susceptibility and as such would result in underestimation of the true magnitude of effect.

## Conclusions

In a unique population controlling for maternal tobacco use, smoking is associated with subclinical cardiovascular disease and secondhand smoke exposure primarily from cigar and pipe use is associated with a greater burden of risk factors for cardiovascular disease. Sex differences are suggested in the correlation between smoke exposure and HDL-C levels, and glucose levels.
